# Comparative Computational Study of Frequency Shifts and Infrared Intensity Changes in Model Binary Complexes with Red- and Blue-Shifting Hydrogen Bonds

**DOI:** 10.3390/molecules30010106

**Published:** 2024-12-30

**Authors:** Sean A. C. McDowell

**Affiliations:** Department of Biological and Chemical Sciences, The University of the West Indies, Cave Hill Campus, Wanstead P.O. Box 64, Barbados; sean.mcdowell@cavehill.uwi.edu; Fax: +1-246-417-4325

**Keywords:** hydrogen bond, frequency shift, infrared intensity, perturbation theory

## Abstract

A computational study of X-H···Y binary hydrogen-bonded complexes was undertaken to examine the red- and blue-shifting behavior of three model X-H proton donors interacting with a series of Lewis bases: Y = NH_3_, NCLi, NCH, NCF, C_2_H_2_, BF, CO, N_2_ and Ne. Two of these proton donors, FArH and F_3_CH, have blue-shifting tendencies, while the third, FH, has red-shifting tendencies. A perturbation theory model for frequency shifts that was derived many years ago was employed to partition the predicted frequency shift into the sum of two components, one dependent on the second derivative of the interaction energy with respect to X-H displacement and the other dependent on the X-H bond length change in the binary complex. The predicted shifts were found to be in good agreement with standard ab initio computations, but they were obtained at much lower computational cost. The change in the infrared intensity of the X-H stretching frequency, expressed as a ratio of complex to monomer intensities, was also investigated, along with its relation to the X-H permanent dipole moment derivative and total induced dipole moment derivative with respect to X-H displacement, and used to rationalize the observed infrared intensity changes in the red- and blue-shifted X-H···Y complexes.

## 1. Introduction

Of all known noncovalent interactions, the hydrogen bond is by far the most well understood and has been the most widely studied for more than a century now. Despite its vintage, it continues to be a fascinating area of research, as can be attested by the number of papers that appear yearly touching on this subject, especially with regard to its cooperative effects or synergies in conjunction with the newer types of noncovalent interactions, such as the halogen and tetrel bond, which have commanded scientific interest over the last decade or so.

As is well known, hydrogen bonding is important for the structure and functioning of biomolecules such as DNA, where base-pairing is a crucial feature of its functionality. The unusual properties of liquid water are also governed, at least to some extent, by hydrogen bonding, which allows each water molecule to act both as a double proton donor and double proton acceptor for other adjacent water molecules. Weakly bound gas-phase species containing protons are also commonly held together by hydrogen bonds. A large body of literature on the characteristics and the importance of the hydrogen bond has accumulated over the century or so since its definition by Latimer and Rodebush in 1920 [1], including refs. [1,2,3,4,5,6,7], which span works published since then and as recently as 2024 [2], illustrating the durability of interest in this important noncovalent interaction.

The hydrogen bond is a relatively weak noncovalent interaction between a proton donor (Lewis acid) containing an X-H bond (where X is an element that is more electronegative than H) and a proton acceptor (Lewis base) Y (which contains a region of high electron density such as a lone pair or a π-electron cloud). A hydrogen-bonded complex can thus be denoted as an X-H···Y interaction that is thought to be mainly electrostatic in nature [2,3,4], possibly with a charge-transfer character [2].

Hydrogen bonding causes the electron-rich region of Y to attract the proton of X-H, causing it to extend, accompanied by a decrease in its vibrational stretching frequency (a red shift). Charge transfer is also thought to occur from the proton acceptor Y to the proton donor, primarily into the antibonding σ*(X-H) orbital, which causes a weakening of this bond and concomitant bond elongation [8]. The red shift is viewed as a clear indication of hydrogen bonding.

However, the X-H stretching frequency can also increase on complexation (a blue shift) in some cases, and there has been much experimental and theoretical evidence for this unusual behavior over the last two and a half decades or so. Three common features, which are the reverse of those associated with conventional red-shifting hydrogen bonds, are notable: X-H bond contraction, blue shift of its vibrational mode and decrease in the infrared intensity of this mode. For example, blue-shifting hydrogen bonds have been identified in several experimental studies of hydrogen-bonded C-H bonds [9,10], as well as in theoretical work [9,10,11,12,13,14,15,16,17,18].

Some authors contend that there are no fundamental differences in the bonding characteristics of red-shifting and blue-shifting H-bonded complexes [12,19], supported by theoretical work which suggests that the blue shifts can be explained by the electrostatic interaction between the proton donor and proton acceptor, coupled with the short-range overlap repulsion [17,18,19]. The competition between hyperconjugation and rehybridization has also been advocated as an explanation for the red- and blue-shifting behavior of hydrogen bonds [20]. The balance between the attraction of the proton to the electron-rich site(s) on Y (favoring bond extension and a red shift) and the opposing effect of a net gain in electron density at the X-H bond region in the presence of Y due to the electron affinity of X (favoring bond contraction and a blue shift) was used to explain the continuous shift from red to blue in hydrogen-bonded complexes [21]. A valence bond approach has also been employed to provide a unified explanation for red and blue shifts observed in both hydrogen- and halogen-bonded complexes [22].

A promising approach to predicting and rationalizing the frequency shifts in hydrogen-bonded complexes is through the application of a perturbative theory of vibrational frequency shifts (originally developed to describe solvent effects in vibrational spectroscopy [23,24,25]). This approach has been useful in predicting red and blue shifts in linear hydrogen-bonded Y···HCl (Y = N_2_, CO, BF) complexes [26], linear FH/FArH···Ng (Ng = He, Ne, Ar, Kr) [27], linear FArH···N_2_/P_2_/CO/OC complexes [28] and linear FArH/FKrH···CO_2_ complexes [29].

The theory involves the application of first- and second-order quantum mechanical perturbation theory and the expansion of the intermolecular interaction potential energy, *U*, as a power series in the normal mode coordinates treating it, along with the cubic anharmonicity, as a perturbation to the harmonic oscillator. The frequency shift is related to the first and second derivatives of *U* with respect to the displacement of the nuclei of a diatomic molecule (or pseudo-diatomic molecule) from its equilibrium separation r_e_ (*U′* and *U″*), and the relative magnitude and sign of these two derivatives determines the type and size of the frequency shift obtained. The bond length change is proportional to −*U′*.

A detailed outline of the perturbative model and its application to some hydrogen-bonded complexes can be found in refs. [26,27], so only a brief outline is presented here. Essentially, the perturbative model approximates the frequency shift on complexation, Δω^model^, for the fundamental absorption band (v = 0 → 1) of a perturbed diatomic oscillator as [25]

*hc*Δω^model^ = (*B*_e_/ω_e_)(*U″* − 3*aU′*) (1)

where *B*_e_ is the rotational constant *h*/(8π^2^*mcr*_e_^2^), *m* the reduced mass, *c*ω_e_ the frequency of the harmonic oscillator and *a* is the cubic anharmonicity constant. The constants *B*_e_, ω_e_ and *a* can be determined either experimentally or by ab initio computations (as in the present work). The interaction potential energy derivatives *U′* and *U″* can be determined numerically by ab initio single-point energy computations on X-H and X-H···Y.

The model can also be used to predict the bond length change (Δ*r*) since it is related to *U*′ by the relationship [24]

Δ*r* = −2*B*_e_*r*_e_ *U′*/(*hc*ω_e_^2^) (2)

By rearranging Equation (2) to make *U′* the subject and substituting for this term in Equation (1), we can reformulate to obtain the more convenient equation below, which partitions Δω^model^ into two components, one dependent on *U″* and the other on Δ*r*,

Δω^model^ = α*U″* + βΔr (3)

where α = *B*_e_/ω_e_ and β = ½(3*a*ω_e_/*r*_e_). As can be seen later, the frequency shifts predicted by this model are generally in good agreement with the shifts computed directly by the standard ab initio analytic second derivatives approach.

Another property related to the X-H vibrational frequency in hydrogen-bonded complexes is the infrared intensity of its stretching frequency, which typically is enhanced on complexation. The intensity of the vibrational fundamental of a diatomic harmonic oscillator X-H is given by the well-known relation for its integrated intensity [30]

I_0_ = [*N*_A_/(12ϵ_0_*c*^2^)] × │∂µ^(0)^/∂r_XH_│^2^ (4)

where *N*_A_ is Avogadro’s number, ∂µ^(0)^/∂r_XH_ is the derivative of the X-H dipole moment with respect to nuclear motion. The relative integrated intensity can then be expressed (or approximated for a pseudo-diatomic, ZX-H) by the equation below

(5) $$\frac{I}{\;I_{0}}=\frac{{\left[\frac{\partial \mu^{\prime }}{\;\partial r_{XH}}\;\right]}^{2}}{{\left[\frac{\partial \mu {}^{\circ}}{\;\partial r_{XH}}\right]}^{2}}=\frac{{\left[\frac{\partial \mu {}^{\circ}}{\;\partial r_{XH}}+\frac{\partial \mu^{ind}}{\;\partial r_{XH}}\right]}^{2}}{{\left[\frac{\partial \mu {}^{\circ}}{\;\partial r_{XH}}\right]}^{2}}$$

where ∂µ′/∂r_XH_ is the total dipole moment derivative of the X-H···Y complex, ∂µ^(0)^/∂r_XH_ is the permanent dipole moment derivative for the X-H monomer and ∂µ^ind^/∂r_XH_ is the total induced dipole moment derivative for X-H···Y. The infrared intensity ratio I/I_0_ is both experimentally and theoretically accessible, and along with ∂µ^ind^/∂r_XH_, upon which it depends, is useful in illuminating the contrasting behavior of red- and blue-shifting X-H···Y hydrogen bonds, as we shall see later.

The main purpose of the current study was to apply the reformulated expression for Δω^model^ (Equation (3)) to predict vibrational frequency shifts and use the infrared intensity ratio I/I_0_ for a range of carefully chosen binary hydrogen-bonded complexes to achieve the following:

(i) Assess the reliability of the Δω^model^ predictions relative to the standard ab initio approach;
(ii) Assess the relative importance of the *U*″ and Δ*r* components to the sign and magnitude of the predicted frequency shift and thereby gain insight into red- and blue-shifting behavior in these complexes;
(iii) Assess the usefulness of the computed ∂µ^ind^/∂r_XH_ values in illuminating red and blue shifting behavior;
(iv) Assess the agreement between the infrared intensity ratios obtained (a) directly and (b) by using ∂µ^(0)^/∂r_XH_ and ∂µ^ind^/∂r_XH_ (both evaluated numerically).

We have included in our model study three different proton donors, denoted symbolically as X-H, paired with a range of proton acceptors Y, chosen such that certain properties, including the frequency shift and the infrared intensity ratio, can be systematically varied and trends identified and rationalized. The proton donors include one with a sizeable positive permanent dipole moment derivative with respect to X-H displacement along the X-H axis (FH), the second with a large negative permanent dipole derivative (FArH) and the third with a small negative permanent dipole derivative (F_3_CH). In fact, the negative ∂µ^(0)^/∂r_XH_ of F_3_C-H is an order of magnitude smaller than for FAr-H.

The proton acceptors include a set of N-bases, NCZ (Z = F, H, Li), whose basicity can be systematically increased by the choice of Z (F < H < Li) and NH_3_. The second set is an isoelectronic series of diatomics YZ (YZ = BF, CO, N_2_, OC, FB), which allow for the systematic variation of the “hardness” of the Y atom, as well as intermolecular dipoledipole electrostatic interactions, varying from attractive (BF, CO) to repulsive (OC, FB) interactions. A π-acceptor (C_2_H_2_) and a “hard” noble gas (Ne) complete the set of proton acceptors. The methodology used in the present study is outlined in the next section.

## 2. Computational Methodology

The Gaussian 09W suite of programs [31] was used to perform all calculations on the monomers and dimers at the MP2/6-311++G(2d,2p) level of theory. All species were optimized, followed by harmonic vibrational frequency calculations evaluated via analytical second derivatives. The absence of any imaginary frequencies confirms that the structures were true minima on their respective potential energy surfaces.

The interaction energies ∆E were calculated as the difference between the energy of the complex X-H···Y and the sum of the energies of the individual isolated monomers (X-H, Y) in their equilibrium geometries. The interaction energies were not corrected for the basis set superposition error (BSSE) since MP2 has a tendency to overcorrect for this error and, in any case, the energetic trends are unlikely to change whether BSSE is taken into account or not. The intermolecular separation (R), selected bond length changes (Δr), harmonic frequency shifts (Δω) of the X-H stretching mode and infrared intensity change on complexation, expressed as the ratio I/I_0_, with I denoting the infrared intensity in the complex and I_0_ the infrared intensity in the monomer, were also computed.

The natural bond orbital (NBO) method [32] was employed at the M062x/6-311++G(2d,2p) level, using the MP2 optimized geometries to determine the change in the occupancy of the antibonding σ*(X-H) orbital on formation of X-H···Y.

In applying the perturbative model to compute the frequency shift of the X-H stretch in the dimers we first assumed that (i) Y remained stationary during the ground-state oscillations of the X-H bond in the dimer and (ii) the internal bond lengths/angles of Y, as well as the X-H bond distances and internal angles, remained fixed (in the complex) at their equilibrium values in the isolated X-H molecule.

The interaction potential energy, *U*, was determined by ab initio calculations on the complex. After optimization of X-H···Y, the X to Y separation was fixed at its equilibrium value in the complex. The optimized X–H bond length in the isolated molecule was computed and taken as *r*_e_ in the complex. For each increment Δ of the X–H bond from its *r*_e_ value, retaining the X–H···Y geometry, the interaction energy was then determined as *U* = E(X–H···Y) − E(X–H) − E(Y), where all three quantities are total energies; Δ was chosen as 0.05 Å, except for the strongly bound FH···NH_3_, FH···NCLi and FArH···NCH dimers, where a smaller displacement value of Δ = 0.01 Å was used to ensure greater numerical stability. Thus, the second derivative of *U* (*U*″) can be determined numerically by finite differences from the computed *U*(*r*_e_), *U*(*r*_e_ + Δ) and *U*(*r*_e_ − Δ) values. After parametrization of α and β for X-H, the frequency shift Δω^model^ could be computed using Equation (3) and compared with the value obtained by Gaussian via its standard ab initio analytic second derivatives routine.

As mentioned in the preceding section, the proton donors studied were FH, FArH and F_3_CH. Each molecule was paired with proton acceptors (Lewis bases) chosen from among NCLi, NCH, NCF, NH_3_, C_2_H_2_, BF, CO, N_2_ and Ne. The computed results for FH···Y, FArH···Y and F_3_CH···Y are in Table 1, Table 2 and Table 3, respectively. Table 4 includes some selected parameters of X-H···Y, as well as the induced dipole moment derivative ∂µ^ind^/∂r_XH_, which was evaluated numerically by finite differences and is arranged so that a comparison of the X-H proton donors (for fixed Y) can be readily obtained.

Table 5 compares the infrared intensity ratio I/I_0_ for complexes of the linear proton donors F-H and F-Ar-H in order to assess its variation in the red- and blue-shifting complexes and also to see how well this particular parameter can be computed using the numerically evaluated dipole moment derivatives, compared with a direct ab initio computation.

The results are discussed in the next section.

## 3. Discussion

In each of the first three subsections we discuss the computed results for XH···Y for each proton donor (Lewis acid) paired with selected members of the proton acceptor (Lewis base) series. We then examine the variation in properties of XH (for fixed Lewis base) in X-H···Y, followed by an investigation of the infrared intensity ratios and trends in the variation of ∂µ^ind^/∂r_X-H_ (which is closely related to the infrared intensity), which, along with ∂µ^(0)^/∂r_X-H_, ultimately determines whether complexation enhances or diminishes the intensity of the X-H stretching vibration. Finally, we sum up our findings in the Conclusions section.

Table 1 shows the parameters computed for the F-H···Y dimers. The interaction energy ΔE generally increases in magnitude going up the table in the order of increasing basicity (or proton affinity), i.e. in the order Ne < YZ diatomics < π-electron base (C_2_H_2_) < N-base, and ranges in magnitude between 2 and 60 kJ/mol. The agreement between the harmonic frequency shifts predicted by the perturbative model (∆ω^model^) and the standard ab initio method (∆ω) is excellent, with percentage differences typically < 5%, except for the marginally stable FH···Ne and FH···FB complexes, where their small frequency shifts are, nevertheless, in fair agreement.

The experimental frequency shifts shown in Table 1 are in reasonable agreement with the theoretical ab initio results, especially for FH···NCH (9% difference), though it should be noted that the harmonic frequency shifts are not directly comparable with the experimental values because the vibrational anharmonicity of the F-H stretching mode has not been taken into account, as well as rotational averaging of the stretching frequency in the complex. Nonetheless, the reasonable agreement between theory and experiment suggests that the theoretical level employed in this study is adequate, especially as the main focus of this work is in identifying and rationalizing the trends for related H-bonded complexes. In this regard, the perturbative model, standard ab initio and spectroscopic data show qualitatively the same trend with increasing binding strength.

For FH···N-base, the intermolecular separation decreases, while ΔE, F-H bond extension, the red shift and associated infrared intensity of the F-H stretching mode and the charge transferred into the antibonding σ*(F-H) orbital increase in magnitude monotonically with increasing Y basicity in the order NCF < NCH < NCLi < NH_3_. The FH···NCLi and FH···NH_3_ interaction energies are similar, though larger in magnitude for NCLi. These complexes are H-bonded via the N lone pair.

By contrast, the T-shaped FH···C_2_H_2_ dimer, which involves H-bonding via the diffuse π-cloud of C_2_H_2_, yields ΔE and other properties smaller in magnitude than the corresponding N-base complexes, but larger in magnitude than the isoelectronic YZ set, except for FH···BF, which has a relatively “soft” B atom capable of significant polarization by the FH dipole moment.

For FH···Ne, the computed parameters are the smallest in magnitude, consistent with the hardness of Ne (i.e., low polarizability) and the fact that this is the most weakly bound dimer.

The interaction energy of the FH···YZ diatomics increases in magnitude in the order FH···BF < CO < N_2_ < OC < FB, i.e., with increasing dipoledipole electrostatic interaction. The permanent dipole moments of B^−^F^+^, C^−^O^+^ and NN are 0.945, 0.269 and 0 D, respectively, at MP2/6-311++G(2d,2p), with the orientation of the YZ dipole reversing for O^+^C^−^ and F^+^B^−^, thereby diminishing the net interaction energy since the dipole-dipole electrostatic forces will now be repulsive for these YZ orientations.

For these diatomics, the H···Y separation decreases in accordance with the decrease in Y atomic radius going from B to C, then increases in accordance with the increasing electrostatic repulsion of the opposed FH/YZ dipoles from O to F.

The partitioning of ∆ω^model^ into contributions from *U″* and ∆r shows that both contributions have the same sign for all complexes and so reinforce each other. For the more strongly bound FH···N-base and FH···C_2_H_2_ dimers, both components are large and negative, yielding large F-H red shifts when added together, with the ∆r component much larger in magnitude. This indicates that the force exerted by the electric field of the Lewis base dominates (since this force is proportional to −∆r) and also causes a “loosening” of the X-H bond (*U″* < 0), corresponding to a decrease in the X-H harmonic force constant.

It should be noted that both *U″* and ∆r decrease in magnitude monotonically from FH···NH_3_ to FH···Ne and for the YZ diatomic series going from Y=B to Y=F, but the “hard” F atom in FH···FB leads to a “stiffening” of the F-H bond (*U″* > 0), corresponding to an increase in the X-H harmonic force constant. Combined with the negative ∆r component of ∆ω^model^, it yields a negligible red shift in FH···FB.

Even though the relatively large positive ∂µ^(0)^/∂r_F-H_ of FH (1.694 D/Å) strongly favors red-shifting behavior in FH···Y, a small blue shift is nevertheless predicted for the weakly bound FH···Ne complex, where the relatively “hard” Ne atom gives rise to a negligible F-H bond contraction and negligible “stiffening” of the F-H bond.

It is evident that charge is transferred from the Lewis base into the antibonding σ*(F-H) orbital for all complexes and the charge transfer increases monotonically with the binding strength (going up the table), but it is essentially negligible for the weakly bound FH···OC, FH···FB and FH···Ne dimers.

Turning our attention to the infrared intensity ratio (I/I_0_), we see that the infrared intensity is always enhanced and the degree of enhancement scales with increasing ΔE. Equation (5) shows that I/I_0_ = (∂µ^(0)^/∂r_F-H_ + ∂µ^ind^/∂r_F-H_)^2^/(∂µ^(0)^/∂r_F-H_)^2^, and since ∂µ^ind^/∂r_F-H_ > 0, for proton donors like FH, which have positive ∂µ^(0)^/∂r_F-H_, I/I_0_ will always be greater than 1, i.e., the infrared intensity of the F-H stretch will always be enhanced on complexation, as is evident from the data in Table 1.

The large negative dipole derivative ∂µ^(0)^/∂r_Ar-H_ for F-Ar-H (−5.032 D/Å) predisposes this proton donor to blue-shifting behavior. This derivative is about three times larger in magnitude than the positive ∂µ^(0)^/∂r_F-H_ of FH. Consequently, the majority of the eight complexes in Table 2 show a blue-shifted Ar-H stretching frequency. However, the most strongly bound representatives of this set, FArH···N-base/C_2_H_2_, exhibit large red shifts, ranging in magnitude between 85 and 980 cm^−1^.

For these dimers, the agreement between ∆ω^model^ and ∆ω is generally excellent, except for the strongly bound FArH···NCH dimer, for which there is a large discrepancy between the corresponding values. However, it is evident that the strong intermolecular forces in this dimer cause a substantial distortion of the FArH molecule, which to some extent loses its identity. For example, the H···N distance of 1.187 Å in FArH···NCH is substantially shorter than the Ar-H bond length of 1.582 Å, suggesting that the proton of FArH is essentially transferred to the N atom of NCH. It should be mentioned that of all the FArH complexes, the F-Ar bond suffers the largest extension (by 0.355 Å) in FArH···NCH. It is therefore not surprising that this complex is the one which shows the greatest discrepancy between the two computational approaches. In fact, NBO analysis suggests the absence of an Ar-H bond and the presence of an H-N bond in FArH···NCH.

The experimental blue shift of 75 cm^−1^ for the Ar-H stretch in FArH···N_2_ is substantially lower than our theoretical value(s) of 150 cm^−1^ and this discrepancy between the magnitudes of the calculated and observed shifts may be partially due to the different matrix shifts of HArF monomer and its N_2_ complex in the low-temperature matrix isolation experiment [37]. The large anharmonicity of the Ar-H stretch may also contribute to this discrepancy.

As is the case for the FH···Y dimers, the intermolecular H···Y distance in the FArH···Y dimers decreases with increasing binding strength (i.e., going up the table), especially as FArH has a relatively large dipole moment.

Because ∂µ^(0)^/∂r_Ar-H_ < 0, the Ar-H bond in FArH tends to contract when the Lewis base approaches (as is evident in the more weakly bound FArH···Ne and FArH···YZ dimers), since this increases the FArH dipole moment, which in turn strengthens the electrostatic dipoledipole interactions in FArH···Y. When FArH is optimized in the presence of a point negative charge, replicating a lone pair or source of electron density, the Ar-H bond contracts in response, showing that the bond contraction is electrostatically driven and does not require the presence of another molecule. For example, optimization of FH and FArH in the presence of a point negative charge of magnitude 0.03 *e* positioned along the extension of the molecular axis at a distance of 2 Å from the proton causes an extension of the F-H bond by 0.0006 Å in the former and a contraction of the Ar-H bond by 0.0016 Å in the latter.

However, the attractive electric force exerted on the proton of FArH by the strong electric field emanating from the N-bases (µ^(0)^ = 3.02 and 2.17 D for HCN and FCN, respectively) is sufficient to overcome the bond-contracting tendency and eventually leads to a “loosening” of the Ar-H bond (*U″* < 0) and red shift of the Ar-H stretching frequency.

As observed for the FH···Y dimers, the infrared intensity of the Ar-H stretch in FArH···N-base is also enhanced, with a large increase in the occupancy of the σ*(Ar-H) orbital, consistent with the Ar-H bond extension and red shift.

The dipole induced in the polarizable π-cloud of the non-polar C_2_H_2_ also seems to be large enough to overcome the Ar-H bond contraction/blue shift tendency; note that the perpendicular component of the C_2_H_2_ polarizability, α_┴_, has a relatively large value: 30.123 a.u. compared to the average polarizabilities of Ne (1.406), Ar (7.520), CO (12.375), N_2_ (10.641), NH_3_ (12.507), NCH (15.159), NCF (16.483) and NCLi (22.409), all computed at MP2/6-311++G(2d,2p).

For FArH···N-base/C_2_H_2_ dimers, the H···Y separation is quite small (1.1–2.0 Å), consistent with their relatively large interaction energies. The Ar-H bond extension and the contributions of *U″* and Δr to ∆ω^model^ (as well as ∆ω^model^ itself) increase in magnitude in the order C_2_H_2_ < NCF < NCH. By contrast, the relatively weaker electric fields produced by the YZ diatomics are insufficient to overcome the bond-contracting/blue-shifting tendency of FArH.

Although C_2_H_2_ also causes Ar-H bond extension/red shift, unlike the situation for the complexes containing the N-bases, the infrared intensity of the Ar-H stretch is diminished by the interaction (I/I_0_ < 1), which suggests that ∂µ^ind^/∂r_X-H_ in the FArH···C_2_H_2_ complex is much smaller in magnitude than in the corresponding N-base complexes. Furthermore, ∂µ^(0)^/∂r_Ar-H_ + ∂µ^ind^/∂r_Ar-H_ must be smaller in magnitude than ∂µ^(0)^/∂r_Ar-H_ (which is negative) for there to be a diminution of infrared intensity on formation of FArH···C_2_H_2_. For example, Table 5 shows that ∂µ^ind^/∂r_Ar-H_ = 17.14 D/Å for FArH···NCF and 9.64 D/Å for FArH···C_2_H_2_, so when combined with ∂µ^(0)^/∂r_Ar-H_ (−5.032 D/Å) yields I/I_0_ = 5.8 (enhancement) for the former and I/I_0_ = 0.84 (diminution) for the latter.

For FArH···Ne, the interaction is mainly due to the polarization of Ne by the large FArH dipole, which induces a dipole moment in Ne and causes the Ar-H bond to contract, because in doing so it increases the FArH dipole moment, which then stabilizes the attractive dipole-induced dipole intermolecular interaction. The contributions of *U″* and Δr(Ar-H) are both positive and similar in magnitude and thus yield a blue shift of the Ar-H stretching frequency. Since the charge transfer from Ne to FArH is negligible, this intermolecular interaction is dominated solely by electrostatics. The infrared intensity is diminished since when the large negative ∂µ^(0)^/∂r_Ar-H_ of FArH is added to the smaller positive ∂µ^ind^/∂r_Ar-H_ for the complex, it yields I < I_0_.

For the FArH···YZ series, FArH···BF was found to be unstable, while for the other four members, as observed for the corresponding FH···YZ series, ΔE decreases in magnitude in the order FArH···CO > FArH···N_2_ > FArH···OC > FArH···FB. In FArH···CO, the dipoles of the individual monomers are favorably oriented (i.e., pointing in the same direction), so this complex enjoys an attractive dipole-dipole electrostatic interaction, augmented by a relatively large induction component due mainly to the polarization of CO by the large FArH dipole. FArH···N_2_ is more weakly bound, since N_2_ does not have a permanent dipole moment, so the electrostatic interaction would be dominated by the weaker dipolequadrupole forces. Consequently, the intermolecular separation increases (by 0.2 Å) relative to FArH···CO.

In FArH···OC and FArH···FB, the dipoles are opposed such that the electrostatic forces are now repulsive, and so the interaction will thus be stabilized mainly by the induction forces, with the repulsion being greater in the latter than the former since µ(FB) > µ(OC). Consequently, the intermolecular separation increases in the order N_2_ < OC < FB.

For FArH···CO, the most strongly bound representative, the contraction of the Ar-H bond (column 4, Table 2) is offset by the transfer of charge (from CO) into the σ*(Ar-H) orbital (last column, Table 2), which yields a small “loosening” of the Ar-H bond (*U″* < 0, column 5, Table 2), thereby yielding a relatively small blue shift.

By contrast, charge is transferred *out* of the σ*(Ar-H) orbital in FArH···N_2_, which augments the bond-contracting tendency of FArH, yielding the largest bond contraction (by 0.0115 Å) and “stiffening” of the Ar-H bond (*U″* > 0), which together give rise to the largest blue shift (150 cm^−1^) for the FArH dimer series. As the electrostatic repulsion increases from N_2_ to OC to FB, FArH and YZ move further apart, leading to a steady decrease in *U″* and Ar-H bond contraction, and, as a consequence, the Ar-H blue shift also decreases.

Turning our attention to the infrared intensity changes for this FArH···YZ series, it is evident that I/I_0_ < 1 for the four dimers. As the monomers come closer together, going from FArH···FB to FArH···OC to FArH···N_2_, the positive ∂µ^ind^/∂r_Ar-H_ increases accordingly, and when combined with the negative ∂µ^(0)^/∂r_Ar-H_, leads to an increasing diminution of I/I_0_ from 0.63 to 0.33 to 0.08, respectively (Table 5). However, I/I_0_ for FArH···CO (0.15) is probably larger than it is for FArH···N_2_ (and contrary to the trend), because a significant amount of charge (0.028 *e*) is transferred into the σ*(Ar-H) orbital, whereas relatively smaller amounts of charge are transferred out of this orbital in the other three dimers (see column 10 of Table 2).

It should be emphasized that since the Ar-H bond contraction/blue shift in FArH can occur even when this molecule is optimized solely in the presence of a negative point charge, charge transfer is not necessary to effect the observed geometric/spectroscopic changes, but these changes may nevertheless be either augmented or diminished by intermolecular charge transfer.

We now consider a proton donor with a much smaller negative ∂µ^(0)^/∂r_X-H_ than FArH. Although F_3_CH is the only non-linear proton donor in our study and also has the largest number of bonds (three) adjacent to the X-H (C-H) bond, the agreement between ∆ω^model^ and ∆ω is still quite good, especially for the more weakly bound complexes, e.g., F_3_CH···Ne and the F_3_CH···YZ series. Since the perturbative model treats X-H as a pseudo-diatomic for proton donors like F_3_CH, essentially ignoring any changes in the bonds adjacent to the C-H bond, then the largest discrepancies between the two approaches would be expected for the most strongly bound complexes, where the largest changes in the adjacent bonds would occur. It is therefore not surprising that the largest discrepancies occur for F_3_CH···N-bases, though the agreement is still reasonable, except for F_3_CH···NCLi, where the perturbative model predicts a negligible C-H red shift of 1 cm^−1^, whereas the standard ab initio method predicts a blue shift of 10 cm^−1^.

This large discrepancy in frequency shifts for F_3_CH···NCLi is due to the relatively large change in the C-F bond length on complexation. The C-F bond extends by 0.0066 Å for this dimer, whereas for F_3_CH···NCH and F_3_CH···NCF, it extends by about 0.0031 Å, and for the weakly bound F_3_CH···N_2_, F_3_CH···OC and F_3_CH···FB dimers, the C-F bond length change < 0.001 Å in magnitude.

We also note the good agreement between the theoretical and experimental C-H frequency shifts for F_3_CH···NH_3_, F_3_CH···N_2_ and especially F_3_CH···CO, which provides further validation for the theoretical level employed in the present work.

As previously noted for the FH and FArH complexes, the intermolecular H···Y distance for the N-base, C_2_H_2_ and Ne complexes of F_3_CH generally decreases with increasing binding strength (going up the table). However, this trend is reversed for the F_3_CH···YZ set, where the interacting F_3_CH and YZ monomers move further apart in the order Y = F < O < N < C < B (going up the table), i.e., the intermolecular H···Y distance correlates with increasing Y atomic size.

As is evident for FArH···Y, the N-bases form the most strongly bound F_3_CH···Y complexes, with interaction energies ranging in magnitude between 14 and 30 kJ/mol. However, the electrostatic interaction between the N-bases and the less polar F_3_CH (with a less negative ∂µ^(0)^/∂r_X-H_) does not cause C-H bond extension in F_3_CH···NCLi/NCH/NCF but only a small C-H bond elongation (≈0.001 Å) in F_3_CH···NH_3_.

The C-H bond contracts, and its vibration is blue-shifted in all eleven complexes shown in Table 3, except for F_3_CH···NH_3_, where the “loosening” of the bond (*U″* < 0) and the small bond elongation combine to give the only red shift. The relatively small bond contraction and blue shift for F_3_CH (compared with FArH) is due to its smaller dipole moment, which induces a much smaller dipole in the proton acceptor Y, which along with the permanent dipole moment of Y, provokes bond contraction in F_3_CH.

It is interesting to compare F_3_CH···NCLi with F_3_CH···NCH, since NCLi is more polar and has a larger basicity (or proton affinity) than NCH and is also closer to F_3_CH than NCH (by 0.17 Å). The NCLi appears to “loosen” the C-H bond (*U″* < 0), leading to a negligible C-H bond contraction and red shift, whereas the relatively “harder” NCH “stiffens” it (*U″* > 0), leading to a much larger bond contraction and blue shift; the charge transfer is negligible in both complexes. By contrast, NH_3_ causes a “loosening” and extension of the C-H bond in F_3_CH···NH_3_, leading to a small red shift of the C-H stretch.

Table 3 shows that for the F_3_CH···N-bases, the C-H bond is increasingly “stiffened” and compressed as the basicity of the N-base decreases in the order NCLi < NCH < NCF; consequently, the C-H blue shift also increases with decreasing basicity for these dimers. Surprisingly, C_2_H_2_, with its diffuse π-cloud, and the hard Ne atom give rise to smaller C-H blue shifts in their complexes with F_3_CH than their NCH/NCF counterparts because the contributions of *U″* and Δr to the C-H frequency shift for these complexes are smaller in magnitude.

Turning our attention to the infrared intensity changes on complexation, it is evident that the C-H infrared intensity change is quite different for each complex. A large enhancement (I/I_0_ = 1.9) for NCLi and large diminution (I/I_0_ = 0.08) for NCH indicates the effect of the much larger polarization of F_3_CH by NCLi vis-à-vis NCH; F_3_CH···NH_3_ shows the least diminished infrared intensity due to the N-bases (I/I_0_ = 0.70).

The diffuse π-cloud of C_2_H_2_ is polarized to a lesser extent than the N lone pairs of the N-bases, leading to a smaller ΔE value, C-H bond contraction/blue shift and a smaller reduction in the infrared intensity (i.e., larger I/I_0_ value).

In F_3_CH···Ne, the low polarizability of the “hard” Ne atom leads to a relatively small C-H bond contraction, which yields a blue shift of 12 cm^−1^ when combined with the positive *U″*. The relatively small ∂µ^ind^/∂r_X-H_ contribution to I for F_3_CH···Ne gives rise to the smallest decrease in intensity (I/I_0_ = 0.76).

Turning our attention to the F_3_CH···YZ series, it is evident that F_3_CH and YZ are furthest apart (at 2.75 Å) when YZ = BF, despite this being the most strongly bound dimer. As mentioned before, the H···Y distance (and interaction energy) decreases monotonically going from F_3_CH···BF to F_3_CH···FB, with the separation in the latter being the closest (at 2.50 Å). The electrostatic dipoledipole interaction would be the largest for F_3_CH···BF, where the dipole moment of BF would tend to extend the C-H bond, but this effect is offset by the bond-contracting tendency of F_3_CH (due to its negative ∂µ^ind^/∂r_C-H_).

It should also be noted that the C-H bond contracts less in F_3_CH···BF than in F_3_CH···CO since more charge is transferred into the antibonding σ*(C-H) orbital in the former (0.012 *e*) than in the latter (0.004 *e*), thereby reducing its contraction in F_3_CH···BF relative to F_3_CH···CO. Thus, the charge transferred into the σ*(C-H) antibonding orbital also diminishes the bond-contracting tendency and decreases from BF to FB.

The balance between the bond-extending and bond-contracting factors explains the variation in C-H bond contraction going from Y = BF to Y = FB. Though the changes are rather small, the C-H bond contraction is at a minimum for BF (0.0003 Å) and increases going to CO (0.0008 Å), where the small CO dipole exerts a smaller bond-extending force on C-H than BF. The C-H bond contraction decreases again for OC (to 0.0006 Å) because the directions of the induced dipoles are now reversed (since the F_3_CH and CO dipoles now oppose each other) and thus diminishes the force on the C-H bond, which drops again to a minimum contraction for FB (0.0003 Å). Table 3 shows that this trend for Δr is also mirrored in the variation of *U″*, so when these two parameters are combined, ∆ω^model^ also follows this trend.

For F_3_CH···YZ, the infrared intensity of the C-H stretching frequency always decreases since ∂µ^(0)^/∂r_C-H_ is negative and ∂µ^ind^/∂r_C-H_ is always positive; therefore, I/I_0_ < 1 (see Equation (5)). Since ∂µ^ind^/∂r_C-H_ decreases in magnitude going from BF to FB (i.e., in the order of decreasing Y polarizability), the combination of decreasing positive ∂µ^ind^/∂r_C-H_ and the negative ∂µ^(0)^/∂r_C-H_ (Equation (5)) results in the largest diminution in infrared intensity for BF and the smallest for FB. Consequently, I/I_0_ increases monotonically going from BF to FB, as shown in Table 3.

Table 4 compares selected properties of the three model proton donors, taken together as a group, complexed to the proton acceptors NCH (a moderately strong polar Lewis base H-bonding via an N lone pair), C_2_H_2_ (a medium-strength non-polar base bonding via the C≡C π-cloud), CO (a moderate base with a small dipole moment) and Ne (a “hard” and weak base). The strength of the intermolecular interactions increases going up the table from the weakly bound X-H···Ne group to the strongly bound X-H···NCH group. The more polar the proton donor, the more strongly it binds to the Lewis base, so the interaction energy increases and the intermolecular separation decreases going from F_3_CH to FH to FArH.

Generally, the more strongly bound FArH/FH molecules are closer to the Lewis bases than their more weakly bound F_3_CH counterparts, with Pauli repulsion significantly limiting the closeness of F_3_CH to the Lewis. Consequently, the C-H bond is always contracted and blue-shifted in the F_3_CH···Y complexes.

By contrast, the shortest H···Y distances are for FArH, which has a much larger dipole moment (µ > 7 D) than the other two proton donors (µ < 2 D). So the complexes of FArH and F_3_CH span the two extremes of binding strengths and intermolecular distances in our model X-H···Y complexes. We shall now consider the properties for each group (with a fixed Lewis base) from the weakest (XH···Ne) to the strongest (XH···NCH) hydrogen bonding interactions.

For the XH···Ne complexes, the binding is due mainly to induction forces, which for the “hard” Ne atom will be small, consistent with the relatively small ∂µ^ind^/∂r_X-H_ and negligible charge transfer values shown in Table 4. This “hard” interaction causes compression of the X-H bond with a concomitant blue shift of its stretching mode in all complexes, and both *U″* and Δr make positive contributions to Δω^model^. The infrared intensity ratio I/I_0_ correlates with the red- or blue-shifting tendency of the proton donor, i.e., enhancement for proton donors with positive ∂µ^(0)^/∂r_X-H_ (FH) and diminution for those with negative ∂µ^(0)^/∂r_X-H_ (FArH, F_3_CH).

For the next more strongly bound series, XH···CO, we generally see shorter H···Y distances, facilitating larger charge transfers from CO into the antibonding σ*(X-H) orbital and the emergence of red-shifting behavior in FH···CO with blue shifts obtained for the other two proton donors. Although charge transfer from CO is largest for FArH···CO, the resulting extension of the Ar-H bond is offset by the large blue-shifting tendency of FArH (consistent with its relatively large ∂µ^ind^/∂r_X-H_ value of 7.71 D/Å). This leads to net bond contraction and blue shift—as mentioned before, the other FArH···YZ diatomics are also blue-shifted.

Turning to the next more strongly bound series, XH···C_2_H_2_, it is evident that the “soft” polarizable C≡C π-cloud of acetylene leads to larger ∂µ^ind^/∂r_X-H_ in these complexes than for the XH···CO analogues, except for F_3_CH···C_2_H_2_, resulting in X-H bond extensions/red shifts for F-H and even for Ar-H in the blue-shifting FArH molecule. The FH···C_2_H_2_ and FArH··· C_2_H_2_ dimers have negative contributions to Δω^model^ from both *U″* and Δr, whereas both the *U″* and Δr components of Δω^model^ are positive for the F_3_CH···C_2_H_2_, yielding the only blue shift/bond contraction for this series. The previously noted infrared intensity pattern that distinguishes between the red- and blue-shifting proton donors is also evident for the XH···C_2_H_2_ group, i.e., I/I_0_ > 1 for FH and I/I_0_ < 1 for FArH and F_3_CH.

Charge transfer from C_2_H_2_ into the σ*(X-H) orbital is relatively significant in FArH··· C_2_H_2_ and FH···C_2_H_2_, but much smaller in magnitude than in the corresponding FArH/FH···CO complexes. This is probably because the charge from the diffuse π-cloud is spread out over the two C atomic centers compared with the single C atom of CO. As evident in all of the F_3_CH complexes, the charge transfer from the Lewis bases, either in or out of the antibonding σ*(C-H) orbital, is always relatively small, consistent with their relatively large intermolecular H···Y distances.

Turning finally to the most strongly bound dimers, XH···NCH, it is evident that ∂µ^ind^ /∂r_X-H_ is significantly larger in magnitude than in the other three series, which is no doubt due to the high polarity of the Lewis base HCN and the closeness of the interacting monomers to each other (0.2–0.8 Å closer than in the corresponding CO analogues), which itself is a consequence of the strong electrostatic intermolecular interactions.

For FArH···NCH and FH···NCH, charge transfer from the N lone pair into σ*(Ar-H) and σ*(F-H) is significant and reinforces the electrostatic force which extends the Ar-H/F-H bond and lowers its vibrational frequency; in fact, NBO analysis indicates a significant charge accumulation between the proton of FAr-H and the N of N≡CH in FArH···NCH, such that a partially covalent H-N bond is predicted between the two monomers.

As observed for the other F_3_CH···Y series, the *U″* and Δr contributions to Δω^model^ are positive, which inevitably leads to C-H bond contraction/blue shift in F_3_CH···NCH. Interestingly, although ∂µ^ind^/∂r_X-H_ for F_3_CH···NCH is significantly smaller in magnitude (<1 D/Å) than for FArH···NCH and FH···NCH, it however has the largest value for the F_3_CH···Y series and yields the largest diminution in X-H infrared intensity of all the complexes (I/I_0_ = 0.08). This large drop in infrared intensity is a consequence of the relatively small magnitude of ∂µ^(0)^/∂r_C-H_ for F_3_CH.

We note that the infrared intensity trend observed for the other X-H···Y dimers is also evident for the X-H···NCH dimers, except for the most strongly bound complex, FArH···NCH, where an enhancement of the Ar-H intensity (by a factor of 5.3) is predicted, whereas only decreases in intensity were noted for the FArH···C_2_H_2_/CO/Ne analogues. This observation is consistent with the rather large ∂µ^ind^/∂r_X-H_ value of 27.16 D/Å, which dominates the much smaller ∂µ^(0)^/∂r_X-H_ value of −5.032 D/Å, leading to an increase rather than decrease in infrared intensity.

The induced dipole moment derivative with respect to X-H bond length change in X-H···Y complexes, ∂µ^ind^/∂r_X-H_, can be used as a measure of the extent of mutual polarization of the interacting monomers. This derivative can be related to the infrared intensity ratio I/I_0_ using Equation (5). Thus, the infrared intensities can be evaluated either directly from the normal modes computed via analytical second derivatives (implemented in Gaussian) or they can be assessed using Equation (5), with the dipole derivatives computed numerically by finite differences.

Table 5 compares the ∂µ^ind^/∂r_X-H_ values for dimers of FH/FArH and the Lewis bases. For each proton donor X-H, the dimers are arranged in order of increasing binding strength (going up the table). It is evident that ∂µ^ind^/∂r_X-H_ increases with binding strength for both the FH and FArH series and this strongly indicates the central role of polarization (which is itself a consequence of the relative polarities of the Lewis acid and Lewis base). Since FArH is more polar than FH, ∂µ^ind^/∂r_X-H_ is larger for complexes of the former than for the latter, as seen in Table 5. It is evident that I/I_0_ follows the same trend as that for ∂µ^ind^/∂r_F-H_ since I/I_0_ is dependent on ∂µ^ind^/∂r_X-H_ (via Equation (5)).

As expected, the “hard” Ne atom yields the smallest ∂µ^ind^/∂r_X-H_ values. For the YZ diatomics, the transition from noncovalent interactions of X-H with the harder (i.e., less polarizable) Y atoms F, O and N in FH/FArH···YZ to the softer (i.e., more polarizable) Y atoms C and B is clearly reflected in the monotonic increase in their corresponding ∂µ^ind^/∂r_X-H_ values, ranging from 0.76 D/Å (FH···FB) to 2.48 D/Å (FH···BF) and from 1.01 D/Å (FArH···FB) to 7.71 D/Å (FArH···CO). The smaller ∂µ^ind^/∂r_X-H_ of 2.08 D/Å for FH···C_2_H_2_, relative to FH···BF, suggests a “softer” interaction of FH with BF than with C_2_H_2_.

For the FH···N-base series, ∂µ^ind^/∂r_F-H_ scales with the polarizability of the N-base in the order NCH < NCF < NCLi; FH···NH_3_ has the second largest ∂µ^ind^/∂r_F-H_ value, since NH_3_ is significantly closer to FH than NCH or NCF in their complexes with FH.

The I/I_0_ ratios were computed by (i) direct evaluation and (ii) via Equation (5), employing numerical dipole derivatives for the FH···Y and FArH···Y complexes. Table 5 shows excellent quantitative agreement between both methods for all of the FH···Y dimer set. Quantitative agreement was also achieved for the more weakly bound FArH···Y complexes (specifically for Y = N_2_, OC, FB, Ne), but only fair agreement for the more strongly bound representatives (i.e., Y = NCF, C_2_H_2_, CO). The poorer agreement for the more strongly bound FArH complexes is probably due to the fact that the dispersion forces (which are not adequately described by Equation (5)) become more significant in these complexes because the interacting monomers are relatively close together (i.e., < 2 Å separation).

Nevertheless, the trends for I/I_0_ with increasing binding strength are qualitatively similar for both FH and FArH complexes, and the two different methods are in qualitatively good agreement.

## 4. Conclusions

1. The frequency shift predicted by the perturbation theory model Δω^model^ = α*U″* + βΔ*r* is generally in near-quantitative agreement with the standard ab initio approach for the three model X-H···Y complexes studied here, except for those complexes like FArH···NCH, where the individual molecules essentially lose their identity on complexation.
2. The *U″* and Δ*r* components of Δω^model^ generally reinforce each other (i.e., have the same sign). When they compete, the net frequency shift (whether red or blue) is invariably relatively small. For example, a small red shift of 1 cm^−1^ (FH···FB) and a small blue shift of 2 cm^−1^ (FArH···CO) were obtained when these components had opposite signs (see Table 1 and Table 2).
3. For complexes with a large red shift (>100 cm^−1^; e.g., FH···NCLi), there is significant charge transferred from Y into the σ*(X-H) orbital, whereas for complexes with a large blue shift (>100 cm^−1^; e.g., FArH···N_2_), charge is transferred out of σ*(X-H). For a less polar proton donor like F_3_CH, the charge transferred in or out of this orbital is usually negligible in magnitude (i.e., <0.005 *e*), except for F_3_CH···BF, where the “soft” B atom transfers (>0.010 *e*) of charge into σ*(C-H).
4. The infrared intensity changes for the X-H stretching frequency can be explained by considering the sign and relative magnitude of ∂µ^(0)^/∂r_X-H_ and ∂µ^ind^/∂r_X-H_. For proton donors with positive ∂µ^(0)^/∂r_X-H_ (e.g., FH), the intensity is always enhanced because ∂µ^ind^/∂r_X-H_ is always positive, whereas for those with negative ∂µ^(0)^/∂r_X-H_ (e.g., FArH and F_3_CH), it can be either enhanced or diminished depending on the relative magnitude of ∂µ^(0)^/∂r_X-H_ and ∂µ^ind^/∂r_X-H_.
5. The intensity ratio, which is an experimentally sensitive and theoretically accessible parameter, was found to be useful in illuminating energetic trends - for example, the increasing diminution of the intensity of the C-H stretch in F_3_CH···YZ with decreasing binding strength, which exhibits a strong, straightforward trend among all the experimentally measurable parameters.
6. The induced dipole derivative ∂µ^ind^/∂r_X-H_, computed numerically by finite differences, was also found to be useful in assessing energetic trends. For F-H···Y complexes, it can be used to reproduce I/I_0_ values with quantitative accuracy compared to a direct evaluation (see Table 5). Consequently, it can be used to accurately predict infrared intensity changes for *any* X-H diatomic on complexation.
7. For FArH···Y, ∂µ^ind^/∂r_X-H_ also gives excellent predictions of I/I_0_ for the more weakly bound dimers (i.e., with Y = N_2_, OC, FB, Ne) but only fair estimates for the stronger analogues (Y = CO, C_2_H_2_).
8. To sum up, the merits of this reformulated perturbative model are as follows:
  (i) The relative contributions of *U″* and Δ*r* to the frequency shift (whether red or blue) can be readily assessed and thus provide insight into red- and blue-shifting behavior for related sets of complexes;
  (ii) Once α and β are parametrized by computing the relevant X-H molecular constants, the Δω^model^ relation can then be used to predict the frequency shifts for a wide range of different Y species in their optimized X-H···Y complexes;
  (iii) The frequency shift can be obtained at much lower computational cost than from a full analytical second derivative calculation (using the Hessian matrix), since it involves only three single-point energy calculations on an optimized X-H···Y complex at *r*_e_, *r*_e_ + Δ and *r*_e_ − Δ to determine *U″*, from which Δω^model^ can be obtained once X-H has been parametrized. This would make it particularly useful in predicting frequency shifts of, say, a relatively small proton donor interacting with a very large molecule, for which a full analytic second-derivative computation of the system would not be feasible.
9. An example of the usefulness of the approach mentioned in 8 (iii) above can be illustrated by considering the C-H frequency shifts for Cl_3_CH···Y(CH_3_)_2_ (Y = O, S, Se) in Table 6, predicted by the model and compared to literature values taken from a recent experimental infrared spectroscopy and theoretical study [40]. The frequency shifts were predicted by the perturbative model using the optimized structure reported in the literature and, as can be seen, the agreement between the model and both the reported experimental and theoretical values is good. Furthermore, it is evident that the Δ*r* term contributes more to the C-H red shifts than does *U″* in all three complexes.
10. This model can be used to reliably predict frequency shifts for binary complexes, and the induced dipole moment derivative can also be used to accurately predict infrared intensity changes for diatomic proton donors, so both should be applicable to a wide range of binary hydrogen-bonded complexes.

## Figures and Tables

**Figure 1:**
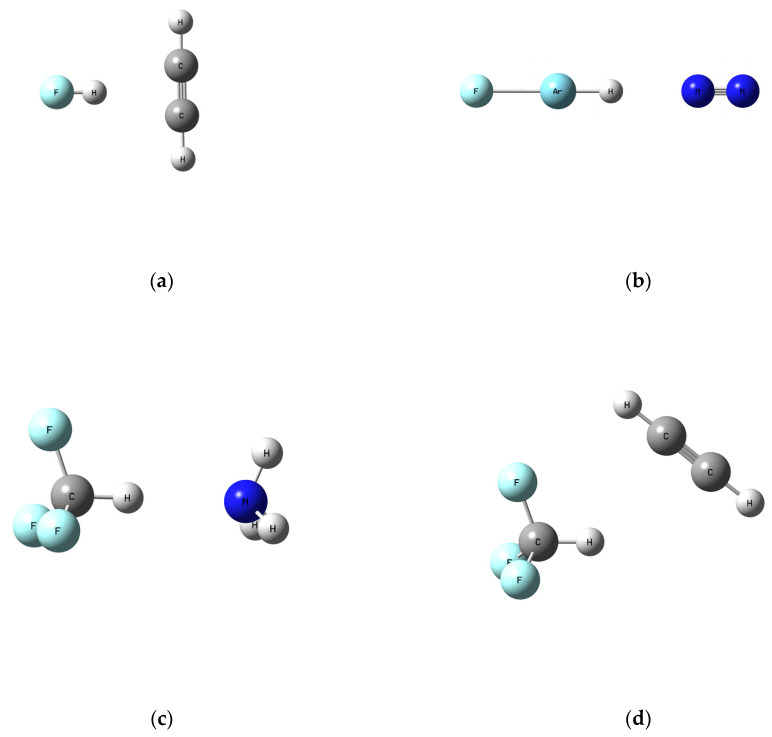
Optimized geometries of some representative X-H···Y dimers: (**a**) FH···C_2_H_2_, (**b**) FArH···N_2_, (**c**) F_3_CH···NH_3_, (**d**) F_3_CH···C_2_H_2_.

**Table 1:** MP2/6-311++G(2d,2p) parameters for FH···Y complexes: the interaction energy (∆E in kJ/mol), intermolecular separation (R in Å), bond length change (∆r in Å), the shift of the harmonic vibrational stretching frequency (∆ω in cm^−1^) and the ratio of its infrared intensity in the complex to its intensity in the monomer (I/I_0_). The change in the NBO occupancy of the antibonding σ*(F-H) orbital on complexation at M062x/6-311++G(2d,2p) is also shown. ∆ω^model^ = α*U″* + β∆r, where *U″* is the second derivative of the interaction potential energy with respect to X-H displacement. All F-H···Y angles are 180° or close to 180°, except for the T-shaped F-H···C_2_H_2_ dimer (see Figure 1). Experimental spectroscopic values for ∆ω are given in parentheses.

Complex	∆E	R(H···Y	∆r(F-H)	α*U″*	β∆r(F-H)	∆ω^model^	∆ω	I/I_0_(F-H)	Δσ*(F-H)
F-H···NH_3_	−53.6	1.7019	0.0325	−209.6	−490.8	−700	−733 (−646) ^b^	12	0.061
F-H···NCLi	−58.6	1.7205	0.0262	−160.4	−395.6	−556	−578	12	0.045
F-H···NCH	−32.7	1.8525	0.0120	−81.4	−181.2	−263	−268 (−245) ^a^	6.9	0.021
F-H···NCF	−30.2	1.8669	0.0107	−70.8	−161.6	−232	−237	6.6	0.018
F-H···C_2_H_2_	−19.7	2.1619	0.0080	−59.5	−120.8	−180	−184	4.9	0.013
F-H···Ne	−2.2	2.1671	−0.0004	6.0	6.0	12	9	1.4	0.002
F-H···BF	−25.0	2.1267	0.0125	−102.2	−196.3	−299	−300	6.4	0.056
F-H···CO	−17.3	2.0751	0.0067	−53.1	−105.7	−159	−157 (−117) ^c^	4.5	0.023
F-H···N_2_	−11.3	2.0772	0.0031	−19.8	−45.3	−65	−69 (−43) ^d^	3.3	0.007
F-H···OC	−7.5	2.1122	0.0012	−2.9	−15.1	−18	−23	2.6	0.002
F-H···FB	−4.2	2.1571	0.0003	3.3	0.0	3	−3	2.0	0.001

Molecular parameters: r_e_(F-H) = 0.9179 Å; ω_e_(F-H) = 4165.3 cm^−1^; µ^(0)^ = 1.8731 D; I_0_ = 127 km/mole; ∂µ^(0)^/∂r = 1.694 D/Å; α = 0.005015; β = −15,100. ^a^ Ref. [33], ^b^ Ref. [34], ^c^ Ref. [35], ^d^ Ref [36].

**Table 2:** MP2/6-311++G(2d,2p) parameters for FArH···Y complexes: the interaction energy (∆E in kJ/mol), intermolecular separation (R in Å), bond length change (∆r in Å), the shift of the harmonic vibrational stretching frequency (∆ω in cm^−1^) and the ratio of its infrared intensity in the complex to its intensity in the monomer (I/I_0_). The change in the NBO occupancy of the antibonding σ*(Ar-H) orbital on complexation at M062x/6-311++G(2d,2p) is also shown. ∆ω^model^ = α*U″* + β∆r, where *U″* is the second derivative of the interaction potential energy with respect to X-H displacement. All Ar-H···Y angles range between 176–180°; FAr-H···C_2_H_2_ is T-shaped. FArH···BF is unstable. Experimental spectroscopic values for ∆ω are given in parentheses.

Complex	∆E	R(H···Y)	∆r(Ar-H)	α*U″*	β∆r(Ar-H)	∆ω^model^	∆ω	I/I_0_(Ar-H)	Δσ*(Ar-H)
FAr-H···NCH	−62.2	1.1870	0.2566	−132.6	−1897	2030	−977	5.2	-
FAr-H···NCF	−51.7	1.4508	0.0652	−199.0	−482	−681	−693	3.4	0.045
FAr-H···C_2_H_2_	−29.6	1.9473	0.0095	−23.6	−70.2	−94	−85	0.57	0.014
FAr-H···Ne	−1.5	2.6666	−0.0015	15.4	11.1	26	27	0.85	−0.002
FAr-H···CO	−21.1	1.9617	−0.0015	−8.9	11.1	2	11	0.15	0.028
FAr-H···N_2_	−16.1	2.1644	−0.0115	64.5	85.0	150	150 (75) ^a^	0.08	−0.017
FAr-H···OC	−5.4	2.2897	−0.0103	62.5	76.2	139	138	0.33	−0.016
FAr-H···FB	−1.8	2.4236	−0.0050	34.9	37.0	72	72	0.63	−0.007

Molecular parameters: r_e_(Ar-H) = 1.3256 Å; ω_e_(Ar-H) = 2151.9 cm^−1^; µ^(0)^ = 7.2127 D; I_0_ = 1149 km/mole; ∂µ^(0)^/∂r = −5.032 D/Å; α = 0.004524; β = −7394. ^a^ Ref. [37].

**Table 3:** MP2/6-311++G(2d,2p) parameters for F_3_C-H···Y complexes: the interaction energy (∆E in kJ/mol), intermolecular separation (R in Å), bond length change (∆r in Å), the shift of the harmonic vibrational stretching frequency (∆ω in cm^−1^) and the ratio of its infrared intensity in the complex to its intensity in the monomer (I/I_0_). The change in the NBO occupancy of the antibonding σ*(C-H) orbital on complexation at M062x/6-311++G(2d,2p) is also shown. ∆ω^model^ = α*U″* + β∆r, where *U″* is the second derivative of the interaction potential energy with respect to X-H displacement. All C-H···Y angles are 180°, except for F_3_C-H···C_2_H_2_, which is almost T-shaped (see Figure 1). Experimental spectroscopic values for ∆ω are given in parentheses.

Complex	∆E	R(H···Y)	∆r(C-H)	α*U″*	β∆r(C-H)	∆ω^model^	∆ω	I/I_0_(C-H)	Δσ*(C-H)
F_3_C-H···NH_3_	−19.6	2.3017	0.0007	−12.4	−6.0	−18	−9 (−12) ^a^	0.70	0.002
F_3_C-H···NCLi	−29.8	2.1949	−0.0002	−3.0	1.7	−1	10	1.89	0.001
F_3_C-H···NCH	−15.2	2.3683	−0.0015	11.2	12.8	24	30	0.08	−0.001
F_3_C-H···NCF	−14.4	2.3633	−0.0017	12.6	14.5	27	32	0.04	−0.001
F_3_C-H···C_2_H_2_	−10.0	2.7340	−0.0009	6.3	7.7	14	17	0.43	−0.001
F_3_C-H···Ne	−1.9	2.6537	−0.0006	6.5	5.1	12	12	0.76	≈0.0
F_3_C-H···BF ^c^	−11.0	2.7515	−0.0003	1.8	2.6	4	8	0.02	0.012
F_3_C-H···CO	−7.8	2.6445	−0.0008	9.7	6.8	17	19 (19) ^b^	0.11	0.004
F_3_C-H···N_2_	−6.1	2.5354	−0.0008	11.9	6.8	19	20 (14) ^b^	0.20	0.001
F_3_C-H···OC	−4.1	2.5136	−0.0006	11.7	5.1	17	18	0.36	≈0.0
F_3_C-H···FB	−2.3	2.5014	−0.0003	9.9	2.6	12	12	0.59	≈0.0

Molecular parameters: r_e_(C-H) = 1.0818 Å; ω_e_(C-H) = 3221.1 cm^−1^; µ^(0)^ = 1.7020 D; I_0_ = 24 km/mole; ∂µ^(0)^/∂r = −0.299 D/Å; α = 0.004751; β = −8530. ^a^ Ref. [38], ^b^ Ref. [39], ^c^ MP2/6-311++G(3d,3p) for F_3_CH···YZ (YZ = BF, CO, N_2_, OC, FB) dimers.

**Table 4:** Comparison of X-H proton donors in X-H···Y complexes (Y = NCH, C_2_H_2_, CO and Ne). The selected parameters for comparison are as follows: (i) H···Y distances, (ii) contributions from *U″* and ∆r to ∆ω^model^, (iii) induced dipole moment derivatives (∂µ^ind^/∂r_X-H_) computed numerically, (iv) infrared intensity ratios (I/I_0_) and (v) the change in the antibonding σ*(X-H) orbital occupancies (computed at M062X/6-311++G(2d,2p)).

Complex	R(H···Y)	α*U″*	β∆r	∂µ^ind^/∂r_X-H_	I/I_0_(X-H)	Δσ*(X-H)	Type of Freq Shift
FAr-H···NCH	1.1870	−300	−1897	27.16	5.3		Red
F-H···NCH	1.8525	−81	−181	2.83	6.9	0.021	Red
F_3_C-H···NCH	2.3683	11	13	0.99	0.08	−0.001	Blue
FAr-H···C_2_H_2_	1.9473	−24	−70	9.64	0.57	0.014	Red
F-H···C_2_H_2_	2.1619	−60	−121	2.08	4.9	0.013	Red
F_3_C-H···C_2_H_2_	2.7340	6	8	0.28	0.43	−0.001	Blue
FAr-H···CO	1.9617	−9	11	7.71	0.15	0.028	Blue
F-H···CO	2.0751	−53	−101	1.95	4.5	0.023	Red
F_3_C-H···CO	2.6445	10	7	0.48	0.11	0.004	Blue
FAr-H···Ne	2.6666	15	11	0.38	0.85	−0.002	Blue
F-H···Ne	2.1671	6	6	0.32	1.4	0.002	Blue
F_3_C-H···Ne	2.6537	7	5	0.11	0.76	≈0.0	Blue

The average dipole polarizabilities of FH, FArH and F_3_CH are 4.130, 25.849 and 16.635. a.u., respectively.

**Table 5:** Comparison of the MP2/6-311++G(2d,2p) infrared intensity ratio (I/I_0_) for F-H···Y and FAr-H···Y dimers evaluated by (i) direct ab initio computation and (ii) numerical derivatives via Equation (5) (see main text and table footnote). The dipole derivatives are in units of D/Å.

Complex	∂µ^ind^/∂r_XH_	I/I_0_ (via Numerical Derivatives)	I/I_0_ (Direct Computation)
F-H···NCLi	4.30	12	12
F-H···NH_3_	4.26	12	12
F-H···NCH	2.83	7.1	6.9
F-H···NCF	2.72	6.8	6.6
F-H···C_2_H_2_	2.08	5.0	4.9
F-H···BF	2.48	6.1	6.4
F-H···CO	1.95	4.6	4.5
F-H···N_2_	1.40	3.3	3.3
F-H···OC	1.06	2.6	2.6
F-H···FB	0.76	2.1	2.0
F-H···Ne	0.32	1.4	1.4
F-Ar-H···NCF	17.14	5.8	3.4
F-Ar-H···C_2_H_2_	9.64	0.84	0.57
F-Ar-H···CO	7.71	0.28	0.15
F-Ar-H···N_2_	3.62	0.08	0.08
F-Ar-H···OC	2.08	0.34	0.33
F-Ar-H···FB	1.01	0.62	0.63
F-Ar-H···Ne	0.38	0.85	0.85

$\frac{I}{\;I_{0}}$ = $\frac{{\left[\frac{\partial \mu {}^{\circ}}{\;\partial r_{XH}}+\frac{\partial \mu^{ind}}{\;\partial r_{XH}}\right]}^{2}}{{\left[\frac{\partial \mu {}^{\circ}}{\;\partial r_{XH}}\right]}^{2}}$; µ^(0)^ is the X-H permanent dipole moment and µ^ind^ the total induced dipole moment in X-H···Y. ∂µ^(0)^ /∂r_XH_ = 1.694 D/Å (F-H); ∂µ^(0)^/∂r_XH_ = −5.032 D/Å (FAr-H).

**Table 6:** MP2/6-311++G(2d,2p) C-H frequency shift ∆ω (in cm^−1^) for Cl_3_C-H···Y(CH_3_)_2_ (Y = O, S, Se) dimers predicted by the perturbative model and compared with the shifts obtained from experimental infrared spectra in N_2_ and Ar matrices at 10K, as well as theoretical computations at MP2/aug-cc-pVDZ [40]. The parameters used in ∆ω^model^ (=α*U″* + β∆r) are α = 0.004815 and β = −9406, and the ∆r values (in Å) were taken from the MP2/aug-cc-pVDZ literature values [40].

Complex	∆r(C-H)	α*U″*	β∆r(C-H)	∆ω^model^	∆ω (N_2_ Matrix)	∆ω (Ar Matrix)	∆ω (Theoretical)
Cl_3_C-H···O(CH_3_)_2_	0.003	−15.7	−28.2	−44	−51	−40	−40
Cl_3_C-H···S(CH_3_)_2_	0.005	−36.2	−47.0	−83	−75	−65	−77
Cl_3_C-H···Se(CH_3_)_2_	0.005	−34.8	−47.0	−82	−83	−74	−81

## Data Availability

Cartesian coordinates of all optimized complexes are available in the Supporting Information file.

## Supplementary Materials

The following supporting information can be downloaded at: https://www.mdpi.com/article/10.3390/molecules30010106/s1.
