# Supplementary material for: Comparative Computational Study of Frequency Shifts and Infrared Intensity Changes in Model Binary Complexes with Red- and Blue-Shifting Hydrogen Bonds
Source: Molecules. 2024 Dec 30;30(1):106. doi: 10.3390/molecules30010106 (PMC11721933; doi:10.3390/molecules30010106)
Supplement: Supplementary file 1 [file molecules-30-00106-s001.zip › molecules-3408089-supplementary.pdf]

Supporting Information

Cartesian coordinates of the optimized geometries of the H-bonded complexes at the MP2/6-311++g(2d,2p) level); MP2/6-311++g(3d,3p) for $F_3CH\ldots YZ$ (YZ = BF, CO, N_2, OC, FB.

1. FH…NH_3

Atom	X	Y	Z
H	0.478591	0.000001	0.000002
N	-1.223295	0.000000	-0.000025
H	-1.592010	-0.813954	-0.470286
H	-1.592013	0.814368	-0.469566
H	-1.592048	-0.000416	0.939994
F	1.428949	0.000000	0.000003

2. FH…NCLi

Atom	X	Y	Z
C	0.043746	1.532153	0.000000
N	0.000000	0.356648	0.000000
Li	0.125167	3.479378	0.000000
F	-0.065916	-2.307149	0.000000
H	-0.044734	-1.363254	0.000000

3. FH…NCH

Atom	X	Y	Z
C	0.033199	1.899433	0.000000
N	0.000000	0.737244	0.000000
F	-0.026992	-2.044997	0.000000
H	-0.021284	-1.115134	0.000000
H	0.065018	2.962797	0.000000

4. FH…NCF

Atom X Y Z

C	-0.207085	-1.089055	0.000000
N	0.000000	0.058312	0.000000
H	0.352002	1.891711	0.000000
F	-0.431453	-2.332470	0.000000
F	0.530398	2.802963	0.000000

5. FH…C_2H_2

Atom	X	Y	Z
F	1.831071	-0.005604	-0.000021
H	0.905255	-0.019304	0.000267
C	-1.229254	0.611187	-0.000011
H	-1.214561	1.673127	0.000019
C	-1.252021	-0.601276	-0.000007
H	-1.282677	-1.662857	0.000008

6. FH…Ne

Atom	X	Y	Z
F	0.003419	1.586293	0.000000
H	-0.064958	0.671231	0.000000
Ne	0.003419	-1.494787	0.000000

7. FH…BF

Atom	X	Y	Z
F	-0.033017	-2.294178	0.000000
H	-0.030127	-1.363740	0.000000
B	0.000000	0.762735	0.000000
F	0.036365	2.021963	0.000000

8. FH…CO

Atom	X	Y	Z
F	0.000000	0.000000	2.166665
H	0.000000	0.000000	1.242064
C	0.000000	0.000000	-0.833058
O	0.000000	0.000000	-1.967963

9. FH…N_2

Atom	X	Y	Z

F	0.000000	0.000000	2.112034
H	0.000000	0.000000	1.191002
N	0.000000	0.000000	-0.886252
N	0.000000	0.000000	-1.999364

10. FH…OC

Atom	X	Y	Z
F	0.000000	0.000000	2.091246
H	0.000000	0.000000	1.172120
O	0.000000	0.000000	-0.940046
C	0.000000	0.000000	-2.078827

11. FH…FB

Atom	X	Y	Z
F	0.000000	0.000000	2.098105
H	0.000000	0.000000	1.179900
F	0.000000	0.000000	-0.977228
B	0.000000	0.000000	-2.253558

12. FArH…NCH

Atom	X	Y	Z
C	-3.238851	0.090515	-0.001292
N	-2.093106	-0.016384	0.001819
H	-0.907688	-0.078688	0.000493
F	3.017259	0.124465	0.000244
H	-4.302714	0.189066	-0.000108
Ar	0.674440	-0.092165	-0.000420

13. FArH…NCF

Atom	X	Y	Z
C	2.543568	-0.007041	-0.000217
N	1.385763	-0.076363	0.002404
H	-0.064955	-0.066802	-0.001295
F	-3.647639	0.063003	0.001298
Ar	-1.455184	-0.027761	-0.001279
F	3.791696	0.064030	-0.000322

14. FArH…C_2H_2

Atom	X	Y	Z
H	0.655410	0.000015	-0.000650
C	2.602595	0.607226	0.000173
H	2.626727	1.670903	0.000186
C	2.602485	-0.607204	0.000183
H	2.626750	-1.670878	0.000165
Ar	-0.679697	-0.000033	-0.000296
F	-2.767203	0.000047	0.000389

15. FArH…Ne

Atom	X	Y	Z
H	-0.712385	0.065626	0.001740
F	2.609974	-0.036932	0.000126
Ar	0.611068	0.025284	-0.000159
Ne	-3.377660	-0.018835	-0.000001

16. FArH…CO

Atom	X	Y	Z
Ar	-0.505143	-0.077528	0.019863
H	0.811767	-0.211936	0.050871
F	-2.561690	0.132565	-0.028826
C	2.762663	-0.411769	0.097380
O	3.889696	-0.528000	0.124227

17. FArH…N_2

Atom	X	Y	Z
N	-2.536743	-0.000015	0.000015
H	-0.372341	-0.003562	0.000188
F	2.969953	0.002138	0.000015
Ar	0.941800	-0.001398	-0.000019
N	-3.650347	0.001371	-0.000011

18. FArH…OC

Atom	X	Y	Z
Ar	0.000000	0.000000	0.964502
H	0.000000	0.000000	-0.350827
F	0.000000	0.000000	2.977174

O	0.000000	0.000000	-2.640554
C	0.000000	0.000000	-3.780058

19. FArH…FB

Atom	X	Y	Z
H	0.317826	-0.073306	0.000553
F	-3.004050	0.043752	-0.000237
Ar	-1.001925	-0.026905	0.000258
F	2.740502	-0.005462	-0.000899
B	4.017750	0.042596	0.001008

20. $F_3CH…NH_3$

Atom	X	Y	Z
H	0.557923	-0.000002	0.000008
C	-0.524545	0.000000	0.000001
N	2.859639	-0.000001	0.000003
H	3.240723	-0.745051	-0.566157
H	3.240726	-0.117783	0.928314
H	3.240722	0.862833	-0.362151
F	-1.005562	1.156901	-0.485322
F	-1.005563	-0.998750	-0.759246
F	-1.005575	-0.158150	1.244563

21. $F_3CH…NCLi$

Atom	X	Y	Z
H	-0.054476	0.000001	-0.000166
C	-1.136091	0.000000	-0.000033
F	-1.622994	1.086399	-0.626513
F	-1.622991	-1.085818	-0.627522
F	-1.622742	-0.000583	1.254149
C	3.319521	0.000000	-0.000035
N	2.140444	0.000002	-0.000079
Li	5.263113	-0.000002	0.000031

22. $F_3CH…NCH$

Atom	X	Y	Z
H	-0.128213	-0.000016	-0.000079
C	0.952047	-0.000002	-0.000068
C	1.429234	-1.085847	-0.627688
F	1.429207	1.086626	-0.626349
F	1.428989	-0.000770	1.254148

C	-3.661668	-0.000002	-0.000032
H	-4.725498	0.000003	-0.000024
N	-2.496490	-0.000007	-0.000043

23. F_3CH…NCF

Atom	X	Y	Z
H	-0.579367	0.000325	-0.000018
C	-1.659488	0.000150	-0.000004
F	-2.135035	-0.627891	-1.086169
F	-2.135005	-0.627022	1.086677
F	-2.135517	1.254656	-0.000499
C	2.953185	0.000010	0.000006
N	1.783939	0.000124	-0.000003
F	4.219958	0.000017	-0.000006

24. F_3CH…C_2H_2

Atom	X	Y	Z
C	0.850444	-0.141986	-0.000034
F	1.574040	-0.441922	-1.086072
H	-0.086137	-0.681530	-0.000161
F	0.623027	1.181433	0.000033
F	1.573783	-0.442036	1.086138
C	-2.563361	0.483362	-0.000009
C	-3.000326	-0.647303	-0.000067
H	-3.397797	-1.631221	-0.000112
H	-2.174261	1.471039	0.000033

25. F_3CH…Ne

Atom	X	Y	Z
H	-0.495119	0.000036	-0.000027
C	0.586115	0.000006	-0.000004
F	1.054286	-0.430707	1.177927
F	1.054321	-0.804768	-0.961952
F	1.054361	1.235453	-0.215959
Ne	-3.148829	0.000012	-0.000009

25. F_3CH…BF

Atom	X	Y	Z
C	-1.110854	-0.000001	-0.000003
F	-1.585453	1.089666	-0.622156
F	-1.585462	-1.083638	-0.632592

F	-1.585458	-0.006024	1.254748
F	-0.028027	-0.000006	0.000008
B	2.723475	-0.000002	0.000002
F	3.987014	0.000000	-0.000001

26. $F_3CH...CO$

Atom	X	Y	Z
C	-1.033172	0.000013	0.000004
F	-1.505603	-0.648125	-1.074465
F	-1.505655	-0.606473	1.098498
F	-1.505666	1.254577	-0.024055
H	0.049155	0.000021	0.000030
O	3.830049	-0.000006	-0.000016
C	2.693633	0.000025	0.000044

27. $F_3CH...N_2$

Atom	X	Y	Z
H	-0.106904	0.000104	0.000049
C	0.975394	0.000035	0.000008
F	1.446478	-0.627096	1.086785
F	1.446394	-0.627716	-1.086448
F	1.446627	1.254728	-0.000368
N	-2.642268	0.000053	0.000028
N	-3.757867	0.000010	-0.000002

28. $F_3CH...OC$

Atom	X	Y	Z
H	-0.133179	0.000007	-0.000100
C	0.949314	0.000001	-0.000010
F	1.419237	-0.339480	1.207852
F	1.419393	-0.876280	-0.897894
F	1.419362	1.215755	-0.309902
O	-2.646830	0.000003	-0.000037
C	-3.784998	0.000001	-0.000007

29. $F_3CH...FB$

Atom	X	Y	Z
H	-0.146030	0.000260	0.000226
C	0.936804	0.000042	0.000041
F	1.405214	-1.085109	-0.629944
F	1.405653	1.087993	-0.624767
F	1.405735	-0.003041	1.254578
F	-2.647462	0.000093	0.000078
B	-3.919409	0.000013	0.000004
